# Secondary Traumatic Stress and Moral Injury in Maternity Care Providers: A Narrative and Exploratory Review

**DOI:** 10.3389/fgwh.2022.835811

**Published:** 2022-05-04

**Authors:** Kathleen Kendall-Tackett, Cheryl Tatano Beck

**Affiliations:** ^1^School of Medicine, Texas Tech University Health Science Center Amarillo, Amarillo, TX, United States; ^2^School of Nursing, University of Connecticut, Storrs, CT, United States

**Keywords:** secondary trauma, moral injury, maternity, labor and delivery, midwives, obstetricians

## Abstract

**Introduction:**

A significant percentage of maternity providers have experienced secondary traumatic stress following a traumatic birth. Previous studies identified it as an issue, but this literature review is 5–9 years old. In addition, the construct of moral injury has significantly increased our understanding of secondary trauma for military veterans. In the wake of COVID-19, this construct also applies to healthcare providers.

**Objectives:**

The present article updates these reviews and compares findings for three groups: labor and delivery nurses, midwives, and obstetricians. The second portion of this review re-examines previously published qualitative research to determine whether moral injury might more accurately describe the experiences of maternity personnel.

**Methods:**

A comprehensive review of PubMed, Scopus, Web of Science, PsychINFO, and CINAHL was conducted in June 2021 using search terms such as compassion fatigue, secondary trauma, moral injury, labor and delivery, nurses, midwives, and obstetricians. Forty articles were identified, but only 16 focused on secondary trauma or moral injury.

**Results:**

Secondary trauma is a significant concern affecting at least 25% of maternity staff. However, some countries have very low rates, which correspond to low rates in childbirth-related trauma in mothers. Secondary trauma can lead to several symptoms, including re-experiencing, avoidance, negative changes in mood and cognitions, and hyperarousal, which can cause significant impairment. As a result, many providers decide to leave the field in the wake of a traumatic birth. The incidence of moral injury is unknown, but a re-examination of previously published qualitative data suggests that this construct, generally used to describe combat veterans, does describe some of what providers have reported. Acts of omission, i.e., failure to stop the harmful acts of others had long-term negative effects on labor and delivery nurses, consistent with data from military samples. Two possible mediators were proposed: hierarchical and gendered relationships in hospitals and agency of care.

**Conclusion:**

The effects of traumatic birth on providers can be severe, including possible psychological sequelae, impaired job performance, and leaving the field. Moral injury expands upon the construct of secondary traumatic stress. This construct better describes the experiences of maternity staff in non-primary roles who witness traumatic births and are often haunted by events that they could not prevent, but often question whether they should have.

## Introduction

Every day, labor and delivery nurses, midwives, and obstetricians witness potentially traumatic events. Mothers and babies can die or be seriously injured. There can be medical emergencies and near-misses. Mothers and their families can be severely impacted by these experiences with sequelae such as depression, anxiety, and posttraumatic stress disorder (1–5). Adverse events are usually swift and unexpected (6). In our many previous articles, we have described the effects of traumatic birth and obstetric violence on mothers. We recognize the importance of that topic, but it is only part of the equation.

In the present review, we focus on the other people who can be harmed by traumatic birth: maternity providers such as labor and delivery nurses, midwives, and in some cases, obstetricians. Maternity staff may be particularly vulnerable because traumatic births starkly contrast with the generally happy nature of their work. The toll on providers is real as this labor and delivery nurse from Beck and Gable's (7) study describes.

Each traumatic birth adds another scar to my soul. Sometimes … I feel like the Picture of Dorian Gray. Somewhere, my real face is in a closet, and it reveals the awful things I've seen during my labor and delivery career. The face I show the world is of an aging woman who works in this lovely place called a delivery room where happy things happen (p. 10).

Generally, hospitals and healthcare systems do not appear concerned with the mental health of their maternity staff, but we argue that it should be a priority. The mental health of their providers directly affects two bottom-line things that organizations do care about: staff retention and quality of care (i.e., customer service). Unaddressed work-related trauma reduces providers' empathy and increases medicalized practice, which both impact cost and patients' experiences. Staff retention also affects profits, but traumatic births can cause providers to leave their jobs. For example, one study of British obstetricians found that 30% of them stopped attending births after they've experienced a traumatic event (6). Given the growing shortage of healthcare workers, this is a serious cause of concern. It also costs organizations tens of thousands of dollars to recruit and train new personnel brought in the replace people who leave. Yet many organizations, including hospitals and healthcare systems, seem blithely unaware of the serious problem simmering just below the surface.

After a traumatic birth, staff can develop secondary trauma. In addition, the construct of moral injury is possibly a more accurate description of what nurses have reported in previously published studies. Until the COVID-19 pandemic, moral injury had mostly been used to describe the experience of combat veterans. With COVID, researchers have also applied it to frontline healthcare personnel who must decide who gets lifesaving treatment when there are not enough resources. A morally injurious event violates people's moral beliefs (8). In other words, they have participated in, or witnessed something they could not stop, but believed was wrong. The trauma field, particularly studies with combat veterans, has recognized that moral injury overlaps with posttraumatic stress but is distinct and leads to a different constellation of symptoms that are not subsumed under the PTSD diagnosis (9).

## Purpose and Methods

The present review focuses on the mental health sequelae of traumatic birth for labor and delivery nurses, midwives, and obstetricians, focusing on secondary traumatic stress and moral injury. Beck previously conducted a systematic review of secondary trauma in nurses (10). The present article extends that work by adding studies conducted in the past 10 years and widening the scope to include midwives and obstetricians, allowing us to compare and contrast their experiences. A literature search was conducted on PubMed, PsychINFO, CINAHL, Cochrane Databases, Scopus, and Web of Science for studies published in the last 10 years. In addition, we conducted a manual search of two journals: *Journal of Traumatic Stress* and *Psychological Trauma*. The search terms were secondary traumatic stress, compassion fatigue, moral injury, labor and delivery, nurses, maternity, midwives, and obstetricians. The search yielded 41 articles, 25 on the construct of compassion fatigue. Formerly, compassion fatigue was synonymous with secondary trauma (11). However, the construct involved to also combine burnout and secondary trauma. We excluded these articles because it was impossible to tease apart the effects of secondary trauma alone. The present review included 16 articles 13 on secondary trauma and 3 on moral injury in maternity personnel). Out of the 16 articles, 6 were qualitative studies, 7 quantitative, 1 used mixed-methods, and 2 were commentaries with specific examples of secondary trauma (often, the author's own experiences). Four review articles were also included for background. Nine studies were on obstetricians, 8 on midwives, 7 on nurses (several studies included more than one group).

**Figure 1:**
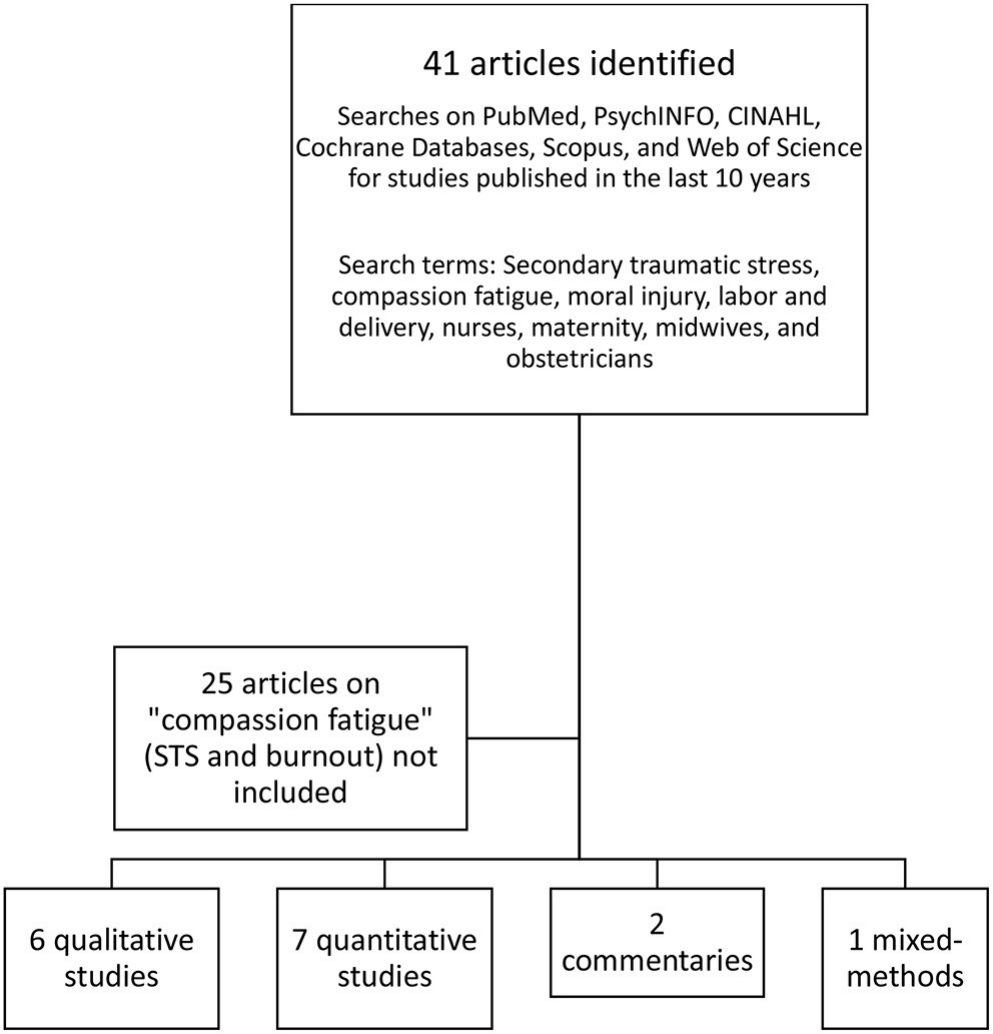
Articles included in review.

Since this literature is somewhat limited, in some sections, (see Figure 1), we included articles on non-maternity nurses and physicians as well as studies of moral injuries military samples, to fill in gaps and suggest directions for future studies.

The second purpose of the present review is exploratory and involves re-examining published qualitative data on secondary trauma maternity providers to determine whether moral injury is a better fit than secondary trauma in describing their experiences. Moral injury was previously described almost exclusively in a military context until the COVID-19 pandemic when articles on moral injury in healthcare providers began appearing in the literature (12). One recent article described COVID-related moral injury in maternity care (13). Previous qualitative studies suggest that the construct of moral injury was in the maternity care literature long before COVID, but without a name or empirically based framework. The purpose of the second section of this review is to re-examine these data in light of this newer framework.

## Secondary Traumatic Stress

When providers witness or participate in traumatic events, they may develop a form of posttraumatic stress disorder known as secondary traumatic stress. Clinically, a traumatic event is defined by the “event criteria” of posttraumatic stress disorder diagnosis in the *Diagnostic and Statistical Manual, 5*^*th*^ *Edition* (DSM-V). DSM-V defines a traumatic event as death or threatened death, actual or threatened serious injury, and actual or threatened sexual violation. These events can be experienced directly or witnessed. For example, Schuster and Dwyer (14), when writing about PTSD in nurses, described a work-related traumatic event as “exposure to traumatic events, overwhelming suffering, and unexplainable loss”. Secondary traumatic stress can develop suddenly and without warning when a provider witnesses traumatic events during birth (10).

### Secondary Traumatic Stress vs. Compassion Fatigue

In some articles, secondary traumatic stress is also called compassion fatigue. Figley (11), the psychologist responsible for much of our understanding of traumatic stress in providers, suggested compassion fatigue as a more friendly and less derogative term. He used the terms secondary traumatic stress and compassion fatigue interchangeably.

Since 1995, the construct of compassion fatigue evolved beyond Figley's original conceptualization to include both burnout and secondary traumatic stress (15). A more recent article further refined the construct by adding compassion satisfaction, which counters the effects of burnout (16). The most recent model describes the balance of job strengths and challenges that determine whether clinicians are willing to stay in the field. Burnout happens gradually, and compassion satisfaction buffers the effects of burnout but does not protect providers from secondary traumatic stress, which can occur with a single incident (17). Interestingly, a recent qualitative study of healthcare providers in the UK found that “burnout” is often a catch-all phrase that includes any mental distress in workers (18). The study participants were frontline healthcare providers who worked during COVID. The researchers found that what was called “burnout” was actually moral injury exacerbated by institutional betrayal, a topic that is relevant to maternity providers and that we explore in the next section.

Unfortunately, from a methodologic standpoint, when the construct of compassion fatigue included both burnout and secondary trauma, findings became more difficult to interpret. Is it burnout, secondary trauma—or moral injury? For example, Stamm's Professional Quality of Life Scale (ProQOL) (19) measures both burnout and secondary trauma. Secondary trauma appears suddenly whereas burnout is a more gradual process. Unfortunately, researchers often combine burnout with secondary trauma when reporting their results, which, in our view, makes findings difficult to interpret. Therefore, for the present review, we chose to focus on secondary trauma alone.

### Incidence

In studies of secondary trauma that only include birth workers, there were two with labor and delivery nurses, three studies of midwives and one of midwifery students, and three studies of obstetricians. We summarize these findings below. Curiously, several of these studies used diagnostic criteria from the 4^th^ Edition of the *Diagnostic and Statistical Manual of Mental Diseases* (DSM-IV) (20), even though the newer criteria from the DSM-V were available in 2013 (9). In the newer criteria, it is no longer necessary to respond to a traumatic event with “fear, helplessness, and horror”. In addition, DSM-V criteria add another symptom cluster: negative changes in mood and cognition. These changes may mean that it is difficult to compare rates of secondary trauma identified with DSM-IV vs. DSM-V criteria.

### Nurses

Labor and delivery nurses have high rates of secondary trauma. Beck and Gable (7) assessed 464 randomly selected labor and delivery nurses from Association of Women's Health, Obstetric, and Gynecologic Nurses (AWHONN). They used the Secondary Traumatic Stress Scale (21) and found that 35% had reported moderate to severe levels of secondary traumatic stress.

Similarly, a study of 144 labor and delivery nurses from the Northeastern U.S. found that 35% had at least mild symptoms of secondary traumatic stress based on their scores on the Secondary Traumatic Stress Scale (STSS). Eleven percent were in the severe range (22). Eighty-five percent of nurses had witnessed a traumatic birth.

### Midwives

Studies on secondary trauma in midwives are international. Most are hospital-based, although some midwives in the Netherlands also practiced homebirth. U.S. studies were of certified-nurse midwives. In reviewing the literature for midwives, two striking findings emerged. (1) The country where midwives practice was directly related to their likelihood of developing secondary traumatic stress. (2) The rates of PTSD in providers were similar to that country's rates of maternal birth-related PTSD. If providers have lower rates of trauma, so do mothers. Conversely, higher rates of PTSD in providers corresponded to higher rates in mothers. We cannot determine a causal relationship between providers' and mothers' experiences. Indeed, both may be a function of wider systemic differences in birthing practices.

#### Lower Rates Traumatic Stress

In Sweden and the Netherlands, rates of birth-related trauma are quite low. For example, a study of 691 Dutch midwives found that only 13% reported a work-related traumatic event, and only 17% of those (2% of the total sample) screened positive for PTSD (23). However, 14% had clinically significant anxiety. The low rates of PTSD correspond to the low rates of PTSD for mothers who give birth in the Netherlands. In a study of 907 women, only 1.2% had PTSD, and 9% identified their births as traumatic (24).

A survey by Wahlberg et al. of 706 Swedish obstetricians and 1,459 midwives found that 7% of obstetricians and 5% of midwives met full criteria for PTSD, and 15% of both groups had PTS symptoms, even though 84% of obstetricians and 71% of midwives had experienced at least one severe event (25). Swedish mothers also report low rates of traumatic birth. In a study of 1,224 women at 1 month postpartum, 1.3% had PTSD (26).

#### Higher Rates of Traumatic Stress

In a U.S. sample of 473 certified-nurse midwives, Beck et al. (27) found that 29% reported high to severe secondary traumatic stress on the Secondary Traumatic Stress Scale, and 36% screened positive for PTSD using DSM-IV criteria. Similarly, a study by these same researchers of 1,373 mothers online and 200 phone interviews in the U.S. Listening to Mothers II survey found that 27% either met full criteria (9%) or had symptoms above the cutoff for posttraumatic stress (18%) (3).

In Turkey, researchers found that 22% of midwifery students (N-465) met full criteria for posttraumatic stress disorder (28). Most of the students had been exposed to potentially traumatic events: 65% had witnessed massive hemorrhage or emergency birth, 68% had witnessed complications during labor, 38% had witnessed mother or infant death, and 88% had witnessed healthcare providers' disrespectful treatment of women. Most had also experienced negative attitudes from other midwives. Rates of maternal birth trauma rates in Turkey were also higher. In a sample of 950 women at 4–6 weeks postpartum, 12% met full criteria for posttraumatic stress disorder (29).

### Obstetricians

The rate of secondary traumatic stress for obstetricians in the Netherlands was nearly identical to the rate that midwives reported in the previous section. A study of 683 Dutch gynecologists found that only 13% had experienced a work-related traumatic event. Of the 13%, 12% of those met the criteria for current PTSD, which is 1.5% for the total sample (30). The authors used DSM-IV criteria. Study participants were all members of the Dutch Society of Obstetrics and Gynecology and included residents, attending, retired, and non-practicing obstetricians/gynecologists.

In the United Kingdom, a study of fellows, members, and trainees of the Royal College of Obstetricians and Gynecologists included an online survey (*N* = 1,095) and 43 in-depth interviews (6). In this sample, 68% reported work-related trauma exposure according to DSM-IV criteria that they responded to with fear, helplessness, and horror. Slade et al. found that 18% of participants had clinically significant posttraumatic stress symptoms, with an additional 13% having subclinical levels. Physicians with PTSD were less satisfied with their jobs, were more emotionally exhausted, took more sick leave, and considered leaving the field.

### Symptoms of Secondary Traumatic Stress

According to the DSM-V diagnostic criteria for PTSD, there are four clusters of symptoms that are necessary for a PTSD diagnosis: re-experiencing, avoidance, negative changes in mood and cognition, and changes in hyperarousal (9). To meet full criteria, you must have symptoms in all four clusters. In addition, symptoms must last for at least 1 month and cause significant impairment. Even if someone does not meet full criteria, they can have symptoms of PTSD. If a person has symptoms for <1 month, they are diagnosed with acute stress disorder (ASD).

Beck (31) conducted a secondary qualitative analysis of three groups of maternal-newborn nurses: certified nurse-midwives, labor and delivery nurses, and Neonatal Intensive Care Unit (NICU) nurses. Across the three groups, 24–29% had secondary trauma symptoms, with intrusive symptoms being the most common. Many recalled vivid images of sights, smells or sounds of births that went wrong. A labor and delivery nurse reported this type of re-experiencing symptoms.

Whenever I hear a patient screaming, I will flashback to a patient who had an unmedicated (not even local) cesarean section and to the wailing of a mother when we were coding her baby in the delivery room. I feel like I will never get these sounds/images out of my head even though they occurred more than 10 years ago [(7); p. 11].

Negative changes in mood and cognitions were the second most prominent symptoms. Nurses reported feeling detached from their emotions and focused on tasks. Guilt, helplessness, and depression were common feelings (31).

The third cluster was the hyperarousal symptoms, including difficulty sleeping, anger and irritability, self-destructive behaviors, and trouble concentrating. NICU nurses felt afraid and guilty that they might have missed something, especially when one of their tiny patients died. Avoidance was also common. All of the nurses in this study avoided things that reminded them of traumatic births, such as a particular room or type of patient. Some avoided television or movie depictions of birth or gore. Some left their work after a traumatic birth (31).

In addition to symptoms of PTSD, providers described other ways that traumatic births affected them. These symptoms listed below would not be included in a diagnosis of secondary trauma, but they exert significant influence over how people practice in the wake of a traumatic event.

### Fear of Litigation and More Medicalized Practice

Fear of being sued can be another part of traumatic births (27, 31). Certified-nurse midwives reported that going through a lawsuit forced them to relive the trauma every day. One nurse-midwife reported that her “soul had died” during the process. One challenge of litigation is that they could not talk with the parents or express sorrow over what happened, which made it difficult to move beyond the experience (27).

### Changes in Practice

A traumatic birth can also cause negative changes in practice. For example, clinicians may become more medicalized (e.g., they order a cesarean section at the first sign of trouble). Or they avoid some practices because they are afraid of the result, as Kerestes (32), a trainee obstetrician, describes.

This is a normal reaction as a healthcare provider: if a cord prolapse occurs, you might hesitate prior to performing your next amniotomy. As the magnitude of the traumatic event increases, the future avoidance may also increase and lead to changes in practice based on one unforgettable encounter (p. 911).

### Loss of Faith in Birth

After witnessing a traumatic birth, some midwives lost their faith in birth (27, 31). This loss of faith can be particularly acute for those who believe that birth is a natural process and not pathology. As this nurse describes, a catastrophic outcome can shake the very foundation of practitioners' beliefs and even cause them to leave the field.

I have many traumatic memories that will be with me always. I went back to graduate school after feeling overwhelmed at the thought of working in labor and delivery until I reached retirement age. Physically and mentally, I knew I would never be able to work that area for another 30 years. I now teach nursing at the college level [(7), p. 11].

Ideals about birthing practice vs. its reality can also provoke another crisis in faith. In a qualitative study from the Northwest of England, 11 student midwives discovered that their training in obstetrics challenged their vision of midwifery (33). Paradigms clashed; the reality of manualized care in a busy O.B. unit vs. a more “woman-led” approach. The students strongly identified with the women and did not like how the hospital treated them. The authors noted that midwifery students operated in a “no man's land” of hospital practice.

### Loss of Empathy

Following a traumatic birth, practitioners may withdraw and even lose their empathy for patients to protect themselves. Maternity nurses reported emotional detachment as a prominent symptom of secondary trauma. Some used numbness as a defense (31). Wahlberg et al. (34) noted that the effects of “severe events,” such as stillbirth, can lessen empathy, make providers feel guilty, and show symptoms of PTSD.

Detachment causes problems because birth practitioners, particularly nurses and midwives, often form relationships with their patients. It's that relationship that increases job satisfaction. However, empathy can be a double-edged sword. It is at the heart of midwifery care, but it also increases the risk for secondary trauma (35). This description for midwives could also be true for labor and delivery nurses.

The features of care that distinguish midwifery from other healthcare professions particularly heightened empathic identification in midwives' relationships with childbearing women, making them vulnerable to traumatic stress. The heart of midwifery care, “being with the women”, has the potential to cause traumatic stress in the midwife in a similar way to how giving birth might do so for the woman (p. 84).

Traumatic birth can blunt practitioners' empathy for their patients, decreasing the quality of care. Some midwives in Beck's (31) study admitted that they got irritable with patients for “whining” about something they perceived as insignificant compared to other women.

### Factors Related to Increased Vulnerability to Secondary Traumatic Stress

All maternity providers face workplace stressors, including exposure to traumatic births, but some providers are more vulnerable to these effects than others. Unfortunately, there are very few studies on vulnerability factors in birthing professionals, but we can draw from studies of other groups of nurses and physicians.

#### Prior Trauma Exposure

There were no studies on the effects of childhood abuse on secondary trauma in birth professionals. However, two recent studies of nursing students suggest that adverse childhood experiences can increase vulnerability. A study of 118 doctors of nursing practice (DNP) students found that an astonishing 50% had experienced at least one adverse childhood experience (ACE), which they hypothesized could affect resiliency (16). A Chinese study of 698 nursing students confirmed this effect. Nineteen percent of their sample reported childhood abuse, which lowered their resilience and ability to withstand stress (36). Emotional neglect had the most negative effect on resilience. Still, other types of family dysfunction, such as poor communication, lack of social support, and negative parent relationship, affected it.

Along these same lines, student midwives were twice as likely to have secondary trauma if they had a history of domestic violence and 90% more likely with a history of trauma (28). In addition, a previous psychiatric diagnosis tripled their risk (28).

#### Cognitions and Worldview

Negative cognitions and beliefs can also increase vulnerability to secondary trauma. For example, if Turkish midwifery students were unsatisfied with their field or wanted to change professions, they were 2.8–4 times more likely to develop secondary trauma (28). Moreover, if they perceived the delivery room as a dangerous place, they were twice as likely when to develop secondary trauma. Worry, anxiety, and self-criticism (trait-negative affect) was also increased vulnerability to secondary trauma in a study of 273 tertiary-care nurses from Western Australia, even after controlling for age, gender, current anxiety and depression, and compassion satisfaction (17).

### Conclusions

Secondary traumatic stress is an unfortunately common experience among birthing professionals and can lead to significant personal and professional impairment. Research in how this affects birthing professionals is limited, and we encourage future studies. Our review highlights areas where the field needs more studies.

The trauma field has broadened its conceptual framework in recent years, recognizing that PTSD and secondary trauma do not describe the full range of symptoms trauma survivors experience. Moral injury encompasses many of the events practitioners describe in qualitative studies following a traumatic birth. It is the focus of the next section.

## Moral Injury

Secondary trauma has been a useful construct for understanding providers' responses to traumatic births, but it is not the entire picture as researchers have discovered. For example, guilt and shame are common responses following traumatic events but are not part of the PTSD diagnosis with DSM-V criteria. In contrast, the construct of moral injury does include guilt and shame and refers to what happens when someone is part of an action that they think is wrong.

Moral injury is a relatively new construct in trauma research and has predominantly been applied to military veterans. In combat studies, the morally injurious act may have been carried out by an individual or group, through a decision made individually or as a response given by leaders (8). Before COVID-19, almost all research on moral injury was conducted with combat veterans. However, in the wake of COVID, healthcare providers have identified moral injury, especially when they are made to comply with policies that violate their moral beliefs (like having to decide who gets treatment and who dies).

A small number of pre-COVID-era articles examined possible moral injury in healthcare providers. For example, a study of 329 hospital-based physicians and nurses examined “moral distress” (15). Inadequate staffing, which providers believed led to inadequate care, increased moral distress. Moral distress increased the risk of burnout, related to turnover, and negatively impacted patient care. In this study, nurses were more likely than physicians to report moral distress. During COVID, there was a sharp increase in articles on moral injury in healthcare providers. The Moral Injury Symptom Scale—Healthcare Professionals, was published in 2020 (37).

### Moral Injury in Maternity Care

As we described earlier, research on moral injury in maternity providers (nurses, midwives, and physicians) is very limited. However, we can apply what we know about moral injury from other populations to birth professionals, which we hope will spur future research. Military research has been particularly helpful. At first glance, research on soldiers does not seem to apply to maternity care providers. However, the comparison is apt when considering hierarchical relationships and chain of command, which are part of the culture in both the military and hospitals.

Moral injury was introduced into the birthing literature during COVID-19 (13). Providers were outraged by policies designed to prevent COVID infections that resulted in mothers laboring alone and mother/baby separation. To capture these reactions, Horsch, as part of a European Union COST action, set up a website for maternity personnel to describe their responses to COVID guidelines (13). This was not a formal study but a collection of first-hand accounts that suggested that providers were experiencing moral injury and were prohibited from providing good care. For example, providers reported that they could not form relationships with the mothers or support breastfeeding. Further, prohibition of birth partners during labor and postpartum.

Central to the concerns of many maternity workers is the disruption of their relationship with the women caused by the introduction of pandemic-related measures.

Some reported that women had forced labor inductions or cesareans against their will. These practices were happening even though they were specifically against the policies established by the World Health Organization, the International Confederation of Midwives, and the International Federation of Gynecology and Obstetrics.

In extreme cases, staff can feel that they have become instruments of inhumane treatment of women and babies—active perpetrators of psychological and physical harm, in complete violation of their moral norms and practice standards …

In conclusion, the unique challenges that the current COVID-19 pandemic poses place maternity staff at risk of engaging in changed practices that may be in direct contravention with evidence; professional recommendations; or, more profoundly, deeply held ethical or moral beliefs and values. This may result in increasing levels of occupational moral injury that need to be addressed, both at an organizational and at a personal level (p. S142).

### Moral Injury in Qualitative Data

Moral injury was identified in healthcare providers during COVID, but what about during non-COVID times? What actions might constitute morally injurious behavior? Richardson et al.'s (38) thematic review of 124 articles synthesized 12 possible definitions of moral injury. In examining these, four seemed particularly relevant to birth.

Injury is brought about by bearing witness to perceived immoral acts, failure to stop such actions, or the perpetration of immoral acts, in particular actions that are inhumane, cruel, depraved, or violent, bringing about pain, suffering, or death of others (p. 577).

Betrayal of what's right by someone who holds legitimate authority in a high-stakes situation (p. 580).

Witnessing or being victim of an act that is perceived to be a gross violation of moral or ethical standards (e.g., killing or injuring civilians, rape, atrocities, betrayal) (p. 581).

Moral injury includes a sense of perceived betrayal unto others, within the self, and/or by an authority figure, which violates personal values and ethics and may result in spiritual and/or psychobehavioral wounds if reconciliation cannot be achieved (p. 581).

We compared these definitions to the nurses' statements from the qualitative portion of Beck and Gable's (7) mixed-methods study of 464 labor and delivery nurses. Beck and Gable originally identified these symptoms as secondary traumatic stress because moral injury was a new construct and only used in studies of combat veterans. Since Beck and Gable's article was published, the literature on moral injury has grown beyond its military confines. As we re-examine these data, we recognize that these nurses describe moral injuries.

The physician violated her.

    A perfect delivery turned violent.

    I felt like an accomplice to a crime.

The doctor treated her like a piece of dirt. After the birth of the baby, he proceeded to put his hand inside her practically halfway up his arm to start pulling the placenta out. She screamed “something is not right. It never hurt like this before." I felt like I was watching a rape” [(7), p. 14].

Speaking about a teen mother, one nurse described the experience (and violation) of a 15-year-old who wanted to give birth without medications (7).

During the delivery, the M.D. was very rough with her perineum and said she wasn't pushing extremely effectively. After two pushes, the M.D. cut a huge episiotomy, and the patient felt it. She screamed in a manner that will always give me chills. The MD said, “this is what happens when you don't get an epidural”. The young mother started crying. It was terrible. He traumatized her and assaulted her. The scream and the M.D.'s comment will always haunt me (p. 9).

This nurse reports re-experiencing symptoms, but she also clearly thought what happened to this young mother was wrong. Another nurse described witnessing a physician manually dilating a patient with each contraction.

My only clear memory is that this beautiful, intelligent, cooperative woman turned into a screaming, mindless animal under his torture.

The nurse blamed herself for not protecting her.

I've never felt so powerless, helpless, or useless in my life. I really feel that I failed her. She was counting on me to help her, and *I let that man torture her* (emphasis added).

The consequences for the nurse were severe.

I feel as sick to my stomach thinking about it today as I did 40 years ago (p. 10).

### Acts of Commission vs. Omission

Studies of soldiers can also be instructive here. Morally injurious events can be acts of commission or omission. Interestingly, the acts of omission (not being able to stop an action) led to more negative effects, including PTSD, depression, and suicidality in a study of 50 Iraq/Afghanistan veterans (39).

This finding helps us understand the reactions of labor and delivery nurses in Beck and Gable's (7) study. They identified a theme in the qualitative portion of their study: “agonizing over what should have been done”.

- Felt powerless because a person in authority was causing unnecessary trauma
- Felt frustrated and angry at physician for not listening
- Feel like I failed my patient
- I should have tried to stop the physician
- My patient was counting on me to protect her (p. 6)

The sequelae for the nurses who witness these events are significant and are generally not addressed. Beck and Gable (7) describe the profound shame and guilt that haunt these nurses as they wonder what they could have done differently to protect mothers in their care. Unfortunately, as often true with soldiers, nurses may not have been able to challenge those in authority, yet they felt like they “should have”, as Beck and Gable describe.

Complicating L&D nurses' helplessness was the guilt that ensued when, at the time, nurses felt that they failed their patients when they did not speak up and challenge/question the obstetricians' practices...

When looking back on the traumatic births, nurses question themselves. What could I have done to prevent this? Did I do everything that I should have done? (p. 10)

A similar theme emerged in a study of certified-nurse midwives: “protecting my patients”. Although certified-nurse midwives are generally primary providers, situations may arise where they are not, such as when physicians take over. Physicians may be rough with patients or babies. Conversely, some reported when obstetricians didn't come and patients died for want of a cesarean that the midwives could not perform (27). NICU nurses also experienced these symptoms and felt angry at the hospital for not doing more to help their patients (31).

### Two Possible Mediators of Moral Injury in Maternity Providers

Moral injury is a new area of study in maternity care. This section explores two possible mediators to moral injury: hierarchical structures, particularly those based on gender and agency of care. We explore these as potential risk factors for moral injury and offer them as possible variables to consider variables in future studies.

#### Gendered Hierarchical Structures in Maternity Care

A persistent theme in birth trauma research is powerlessness, helplessness, and an inability to stop abusive behavior. This raises the question of why providers feel powerless and who is more likely to feel this way? When providers blame themselves for not protecting their patients, how much could they have realistically done to change the situation? Long before researchers considered moral injury in healthcare providers, some theorized about what could be contributing to the power differential, particularly between physicians and nurses. Shay's (40, 41) original conceptualization of moral injury is relevant here where moral injuries combine hierarchical power, betrayal of trust, and a high-stakes situation. McGibbon et al. (42) connected moral distress to nurses' lack of power in a hospital gendered hierarchy. McGibbon notes that the gendered hierarchy of hospitals still exists, even though more women in medicine and more nurses are male.

Nurses' moral distress has been linked to their constrained moral agency as a consequence of their relatively low status in the institutional hierarchy, intraprofessional conflict, the frequent lack of needed resources, and the corporatization of the health care system … [(42), p. 1355].

This historicity is exactly why gender relations between nurses and doctors are not somehow equilibrated with the increase in women in the profession of medicine, or by such things as advances in the status of women's financial remuneration at work. According to Smith, there is a gender subtext of ruling relations that has its historical roots in patriarchy …

The historical development of nursing as a predominately female profession and medicine as a predominately male profession situates nurses in a culturally devalued role, even if currently there are many women in the profession of medicine. *Nurses' everyday practice is articulated to this historical power relationship with medicine and their location in the institutional hierarchy, regardless of present-day efforts to equalize power imbalances* [(42), p. 1368]. Emphasis added.

#### Agency of Care: Primary vs. Secondary Providers

A related theme is whether being “in charge” vs. in a support role influences symptoms of moral injury or secondary trauma. As we learned under COVID, any provider, including physicians, can be forced to do things they think are wrong when hospital policy supersedes their authority (13). However, being in charge may influence both trauma symptoms and moral injury under normal circumstances. For example, an article about risk factors for secondary trauma in midwives speculates whether the agency of care protects midwives sense of wellbeing (35).

Does autonomous midwifery practice increase the risk of developing secondary traumatic stress because of its emphasis on intimate and mutual relationships with childbearing women, or provide protection because, as the primary caregiver, the midwife is in a position of greater control over the birth situation? (p. 85)

When examining the literature reviewed in the previous section, there was a striking consistency in the causes of secondary trauma for obstetricians and midwives. For them, traumatic events are often medical. For example, a study of Dutch obstetricians found that the most frequently reported traumatic events are fetal or neonatal death, shoulder dystocia, and infant resuscitation. Other stressors include missing a diagnosis, doubting a medical decision, or feeling that they could not help the patient (30). Similarly, 706 Swedish obstetricians and 1,459 midwives identified infants dying or being severely injured during delivery, maternal death or near-miss, or other events such as violence or threat as the primary causes of secondary trauma (25).

Similarly, Beck et al.'s (27) study of 473 certified-nurse midwives reported that the most common causes of secondary trauma were fetal/infant demise, shoulder dystocia, and infant resuscitation (27). A sample of 691 Dutch midwives reported that the most stressful events were missing a diagnosis, death or mother or child, life-threatening complications, doubting a medical decision, feeling helpless, and delivering bad news (23). Interestingly, midwives in primary care (at home or birthing centers) were at higher risk for PTSD and depression vs. those who were in secondary and tertiary care in hospitals, under the supervision of obstetricians. The midwives in tertiary care are still in charge of births but have backup physicians if something goes wrong.

A study of midwifery students in Turkey found that events such as massive hemorrhage or emergency birth, mother or infant death as events related to secondary trauma (28). Interestingly, although 88% of student midwives had seen disrespectful care of women, it was not related to secondary trauma. But could it be related to moral injury, which Bingol et al. did not measure in their study?

Labor and delivery nurses can also be traumatized by medical events (22). In addition, not being in charge may also make them susceptible to moral injury *because of the actions of other clinicians*. In Beck and Gable's (7) study, a nurse describes nurses' reactions to an unavoidable medical tragedy vs. avoidable and unnecessary harm.

Traumatic deliveries are much easier to handle and cope with when they are unavoidable. What causes anxiety and stress to nursing staff is when they feel powerless and helpless because another person in authority is causing unnecessary trauma to the patient and infant (p. 10).

An important theme in the Beck and Gable (7) stories was the inability to stop what was happening—acts of omission. Realistically, most of the time, these nurses could not stop what was happening, but they thought they should. These findings are consistent with studies on veterans showing that acts of omission, when they failed to stop an action, had worse effects than acts of commission (8).

### Symptoms

Researchers continue refining the unique contribution of moral injury to symptomatology. Some symptoms of moral injury overlap with those of PTSD, but others are distinct. PTSD leads to a sense of fear and danger, and moral injury leads to anger, guilt, shame, and disgust. When treating a client with moral injury, if mental health practitioners only consider PTSD, they may see guilt, shame, and anger as pathology if it co-occurs with PTSD (43). However, those reactions are appropriate responses to moral injury. Below are the more common symptoms of moral injury.

#### Shame and Guilt

After a morally injurious event, people may condemn themselves, feel betrayed, and lose their faith, sense of meaning, and trust in others (44). Wahlberg et al. (34) noted that severe events, such as stillbirth, can lessen empathy, make providers feel guilty, and show symptoms of PTSD. Their study was a qualitative interview of seven obstetricians and seven midwives in Sweden who had participated in their earlier study about the response to a severe event. Their previous study found that 28% of midwives felt guilty about something they did wrong compared to 47% of obstetricians (25).

A qualitative study of three practitioners who experienced guilt following the death of an infant found that they felt morally responsible for the outcome even though it was not their fault. The study's authors noted that providers need to have their guilty feelings acknowledged rather than having practitioners attempt to take their guilty feelings away (45). Failing to acknowledge practitioners' guilty feelings precludes self-forgiveness, which can impair practitioners' recovery.

#### Self-Esteem, Relationships, and Worldview

Following a morally injurious event, people can see themselves as defective or weak. In extreme cases, they can see themselves as unlovable, and that what they did, or failed to do, makes them unforgivable since they did not stick up for what they believe in. They may no longer trust other people, key relationships, institutions, or organizations. A person who has experienced moral injury no longer believes in a just world or that people were naturally good (46).

Chesnut et al. (44) examined the impact of self-directed and other-directed moral injury on social wellbeing in a sample of 9,566 American veterans. The central concept was, do you blame yourself or others for the event? Those who blamed themselves were significantly less social than those who blamed others. The more they blamed themselves, the less they socialized with others. Guilt and shame may account for some of this effect. The person feels too ashamed to be around other people will limit their social activity. Other-directed moral injuries also caused problems and were related to a steeper decline in social wellbeing.

#### Institutional Abandonment

Another factor can could contribute to symptomatology is institutional abandonment in the form of no recourse for those who witness traumatic births. Nurses who witness traumatic births often find that there is no place for them to report bad behavior by medical staff. And they often experience severe consequences (including firing) if they attempt to report an incident (18). In short, there is no accountability for providers who cause harm. Nursing staff may perceive that hospitals and health systems care more about protecting medical providers, and themselves from liability, than they do about patient care, which they perceive as a betrayal of their duty of care.

#### Posttraumatic Stress Disorder

As we previously described, researchers continue to explore the complex relationship between moral injury and PTSD, and many trauma survivors have both. The combat literature is helpful here too. A study of 182 combat veterans demonstrated the temporal relationship between moral injury and PTSD symptoms, with moral injury preceding trauma symptoms (47). They found that when people had high self- and other-directed moral injury at 1 month, they had more PTSD symptoms at 6 months. Self-directed moral injury includes statements such as “I am ashamed of things I did or saw.” “I punish myself for things I did/saw and neglect my personal safety.” Self-directed moral injury was linked to the PTSD symptom cluster “negative changes in mood and cognitions” and predicted worse PTSD symptoms.

In a study of 258 combat veterans, moral injury predicted depression, anxiety, suicidality, PTSD, and hazardous alcohol use (48). Another study of soldiers found that moral injury increased religious and spiritual struggles, anxiety, and PTSD (49). More ominously, people with both PTSD and moral injury were more likely to have attempted suicide rather than just thinking about it (50).

This symptom pattern can also be true for non-maternity healthcare providers who provided care during COVID. The study included 3,006 Chinese non-maternity physicians and nurses (12). Using the Moral Injury Symptom Scale—Health Professionals (37), Wang et al. found that 41% of physicians and nurses reported moral injury. They were more likely to report moral injury if they provided direct care to COVID patients, but all providers were susceptible even if they did not provide frontline care. For all of them, moral injury related to depression, anxiety, low wellbeing, and burnout.

#### Loss of Faith

Religious or spiritual struggles are common after morally injurious events. Religious coping can be both adaptive (feeling God's forgiveness) or maladaptive (feeling abandoned by God). People who experience a moral injury may reject previously held religious beliefs, report spiritual distress, and feel unforgivable, which increases their suicide risk. They may be more cynical about their beliefs, feel abandoned by God, question their purpose, and believe their actions violated their beliefs and ethics (51).

A recent study involved a Veterans Affairs' chaplain and psychologist in a treatment model for moral injury (43). It was a 12-week, 90-min group intervention to reduce the effects of moral injury. The 40 veterans had an opportunity to explore the religious and spiritual dimensions of their military experience and personal life. The idea was that the onus of warfare does not lie solely on the individual but the general citizenry, so the emphasis is on sharing their stories publicly. They conducted a community healing ceremony in the Veterans Affairs' chapel. The community bears witness to their stories and shares the responsibility. The study had an 80% participation rate. Participation in this program lowered symptoms of moral injury, but a larger trial of this method is needed.

#### Leaving the Field

Following a traumatic or morally injurious event, providers may leave the field. A study of 144 labor and delivery nurses found that nurses with PTSD (with a score of 38 or higher on the Secondary Traumatic Stress Scale) were significantly more likely to consider leaving their jobs, calling in sick, or requesting another assignment (22). Beck and Gable (7) also found that labor and delivery nurses said they were leaving (or left) the field to protect their mental health. A qualitative study of 7 obstetricians and seven midwives found that some have extreme reactions to severe events, including leaving the field or even suicide (34).

Leaving the field was also true for non-maternity healthcare providers. In a study of 329 hospital-based physicians and nurses, 49% of nurses and 28% of physicians left or considered leaving when they scored high in moral distress (15). The highest incidence of moral distress occurred at 6–10 years in the field for both physicians and nurses. The group with the highest psychological health were those who had worked for more than 21 years. Nurses had significantly higher levels of both moral distress and burnout.

## What Helps After Witnessing a Traumatic or Morally Injurious Birth

Witnessing a traumatic birth can devastate providers, potentially causing significant impairment in their personal and professional lives. The possible responses following a severe event are dropping out, surviving, or thriving. Thriving would be the goal of any successful treatment. Although the literature is relatively new, maternity providers have spoken out about what they believe would help them recover. Quite consistently, they spoke about the importance of peer and workplace support.

### Peer Support

Research participants identified support from colleagues as key to recovering from a traumatic birth. A study of 706 Swedish obstetricians and 1,459 midwives found that collegial acceptance was extremely important for helping them regain their professional self-esteem following a critical event (25). For example, one obstetrician reported receiving “absolution” for what they did right, with justified critique for what they did wrong, quite consistent with a moral injury framework. In contrast, avoidance or silence from colleagues negatively affected their recovery. The same was true in a study of 144 labor and delivery nurses who identified co-workers as the most important source of support in the aftermath of a traumatic birth followed by support from family and friends (22).

#### Lack of Peer Support

The lack of peer support also had a notable effect on maternity providers. In their study of certified nurse midwives, Beck et al. (27) found that certified-nurse midwives experienced the full range of symptoms following traumatic births (under DSM-IV criteria). In their qualitative data, the theme “Circling the Wagons” emerged. Some midwives reported that colleagues' support helped them cope following a traumatic birth. However, midwives felt abandoned and betrayed if colleagues did not support them. In addition, when they worked with unsupportive physicians, they reported that their work environment was toxic or unsafe.

Wahlberg et al. (25) noted that for both obstetricians and midwives, guilt; negative reactions from the parents; insufficient support from management, colleagues, friends, and their partner; and negative experiences from debriefing caused significant negative reactions. Obstetricians with partial PTSD were more likely to stop work on the delivery unit, whereas midwives were more likely to call in sick.

Because guilt is common following a traumatic birth, obstetricians suggested that support without blame would help. In Slade et al.'s (6) study of obstetricians and gynecologists, 91% indicated they would like workplace support after experiencing traumatic events. There were three general recommendations:

1) Having someone available to talk about the event shortly after it happened. A senior colleague or dedicated team. Possible time off after a traumatic event.
2) Training providers about trauma must be regular and mandatory. The most beneficial training would include how to manage factors that compound the traumatic experience (e.g., attending coroner's court).
3) Creating a supportive rather than blaming obstetric culture. Destigmatize the need for help and support after a traumatic event.

It did not help when colleagues ignored the event, or when providers were given minimal or “flippant” support. It was also harmful when colleagued criticized or gossiped about them because of the event. Obstetricians reported that open and honest discussion is more helpful than being expected to “carry on”. After a traumatic event, hospitals should normalize and routinely provide support following traumatic births.

### Reframing Beliefs

In PTSD treatment, the focus is often on generating more accurate beliefs about the event. In contrast, in moral injury, interventions primarily focus on the event's moral implications for clients' values without trying to revise clients' descriptive understanding of the event (52). One linguistic clue is the word “should”. “I should have known ….” PTSD treatment emphasizes understanding trauma survivors' beliefs about events they were exposed to. They may draw moral conclusions about themselves, and others based on inaccurate understanding of events. As a result, providers may be overestimating how much they could have done. The goal in therapy is to clarify these underlying beliefs. If trauma survivors can change their beliefs about the event, they may recognize that they did all they could under the circumstances. Reframing beliefs about themselves can be to help them reconnect to others. Farnsworth (52) recommends that therapists emphasize the process of acceptance and reinstatement of valued behaviors, with less effort on challenging the way clients describe the event. The goal is not to change the value judgments but to enhance the client's acceptance of past violations (including taking responsibility for mistakes) and act with values-consistent behavior moving forward.

### Making Meaning of the Event

Many of the beliefs that shape clients' worldview are drawn from religious teachings, cultural traditions, and sociopolitical movements (52). If they're incorporated into treatment, these beliefs can help clients make meaning of the event, which is an important part of healing. Unfortunately, if clients believe that they did wrong, making meaning can be substantially more difficult. They may want to punish themselves, and they have difficulty forgiving themselves or others.

As noted in the previous section, many feel profound guilt and shame. As a result, some lost their faith in the system. Previously, they believed that nothing bad would happen if they worked hard and did their best. Trauma can also affect the just-world belief: “Good things happen to good people. If something bad happened, it must be because I am bad.” They now have a harsher view of the world and believe they will never be the same again.

Part of the healing process is being able to talk about the event with colleagues. It can also help if providers can talk with, and even apologize to the woman or her family. Unfortunately, if there is litigation, that is not always possible. Organizations, such as hospitals or departments, can help increase resilience in their providers if they respond to a traumatic birth with flexibility, trust, and confidence (34). Cognitive-processing therapy can help reframe beliefs; people acknowledge their hopes for a morally just world while recognizing that the world is morally imperfect (52).

However, it is not all about beliefs. There are objective wrong, harmful, and egregious actions in some cases. Unfortunately, there is no mechanism for reporting or accountability in most current hospital settings. Nurses implied this in Beck and Gable's (7) study; there was no recourse, which compounded the nurses' feelings of helplessness. For birth to be safe for patients and practitioners, there must be accountability for harmful or abusive behaviors. Presently, there is a conspiracy of silence between abusive practitioners, witnesses, and the institutions they serve.

In the present time, for practitioners who witnessing harmful acts, contextualizing events can help. For example, it helps soldiers when they acknowledge the unique aspects of military culture, including chain or command, and difficulties that surround making split-second decisions. Similarly, military medics often need to make split-second decisions about who gets life-saving treatment and who does not. In addition, they may be troubled by those they were forced to leave behind. Maternity care also can mean split-second, life-and-death decisions. Context can help practitioners put their actions and the actions of others in a broader framework. It can help them realize that they could do nothing to change the outcome. Recognizing the hierarchical nature of hospitals may also help nurses, in particular, see that they could not have realistically intervened, which may help them move forward.

## Conclusions

Secondary traumatic stress and moral injury affect a high percentage of maternity personnel. The conditions can impair providers in both their personal and professional lives. As a result, some will leave the field to preserve their mental health. Given the growing shortage of healthcare providers, this is an urgent challenge. This article reviewed the literature on secondary trauma in labor and delivery nurses, midwives, and obstetricians. We also re-examined previously published qualitative data within the framework of moral injury. The main findings from this review are as follows:

1) *Secondary trauma is remarkably common in many countries worldwide and can negatively affect both staff retention and quality of care*. Our current estimates are that it affects 25–35% of the workforce. That is a troubling statistic and one that health institutions have largely ignored.
2) *Rates of birth-related secondary trauma differences by country*. For example, both Sweden and the Netherlands have very low rates. In contrast, rates in other industrialized countries, such as the U.S. or Turkey, are much higher.
3) *Rates of secondary trauma for providers mirrors rates of birth-related trauma for mothers*. In countries like Sweden and the Netherlands, the rates are low for both. However, in countries like the U.S. and Turkey, where secondary trauma is common, the rates for providers are similar to the rates for mothers. This finding suggests that providers' mental health is important and addressing it will positively impact on mothers' experiences.
4) *The incidence of moral injury in maternity providers is unknown*. It was first specifically mentioned during COVID (13). However, previously published qualitative data suggests that it has been an issue in maternity care long before COVID-19 and was identified as secondary trauma. The trauma field has learned to differentiate moral injury and PTSD in combat veterans. We suggest that this model can also be useful for maternity providers.
5) *Many evidence-based trauma treatments are available, but fewer options specifically address moral injury*. However, the current literature suggests that peer and institutional support are critical for providers recovering following a traumatic birth. When that support is absent, healing is impaired. Unfortunately, at that point, some providers leave the field.
6) *When providers have a chance to process the events with supportive colleagues and reframe events to understand that they could not have changed the outcome, healing is possible*. Likewise, when practitioners make mistakes, the best way forward is to learn from them and recognize that they did the best they could under difficult circumstances.

In summary, work-related trauma can deeply affect maternity-care providers. If not addressed and the practitioners are not supported, these events can lead to serious physical and mental health issues for providers and possibly impair the care they provide. Healing is possible for everyone affected by traumatic births, but the first step is acknowledging that these events occur and can directly affect staff. Moving forward, the mental health of all maternity care providers needs to be a priority.

## Author Contributions

KK-T conducted the original review of articles, drafted the article, and conceptualized the reinterpretation of secondary trauma literature as possibly describing moral injury. CB was the author of the original studies included in the moral injury section, reviewed and edited the manuscript, and suggested articles to include. Both authors contributed to the article and approved the submitted version.

## Conflict of Interest

The authors declare that the research was conducted in the absence of any commercial or financial relationships that could be construed as a potential conflict of interest.

## Publisher's Note

All claims expressed in this article are solely those of the authors and do not necessarily represent those of their affiliated organizations, or those of the publisher, the editors and the reviewers. Any product that may be evaluated in this article, or claim that may be made by its manufacturer, is not guaranteed or endorsed by the publisher.
